# Simulations of the human heat balance during Mount Everest summit attempts in spring and winter

**DOI:** 10.1007/s00484-023-02594-1

**Published:** 2023-12-19

**Authors:** Krzysztof Błażejczyk, George Havenith, Robert K. Szymczak

**Affiliations:** 1 Institute of Geography and Spatial Organization, Polish Academy of Sciences, Twarda 51/55, 02-818 Warszawa, Poland; 2 Environmental Ergonomics Research Centre, Loughborough School of Design & Creative Arts, Loughborough University, Loughborough, UK; 3 Department of Emergency Medicine, Faculty of Health Sciences, Medical University of Gdańsk, Gdańsk, Poland

**Keywords:** Human heat balance, Hypothermia risk, Mountaineering, High altitude, MENEX_HA model, Mt Everest

## Abstract

The majority of research dealing with the impacts of the Himalayan climate on human physiology focuses on low air temperature, high wind speed, and low air pressure and oxygen content, potentially leading to hypothermia and hypoxia. Only a few studies describe the influence of the weather conditions in the Himalayas on the body’s ability to maintain thermal balance. The aim of the present research is to trace the heat exchange between humans and their surroundings during a typical, 6-day summit attempt of Mount Everest in the spring and winter seasons. Additionally, an emergency night outdoors without tent protection is considered. Daily variation of the heat balance components were calculated by the MENEX_HA model using meteorological data collected at automatic weather stations installed during a National Geographic expedition in 2019–2020. The data represent the hourly values of the measured meteorological parameters. The research shows that in spite of extreme environmental conditions in the sub-summit zone of Mount Everest during the spring weather window, it is possible to keep heat equilibrium of the climbers’ body. This can be achieved by the use of appropriate clothing and by regulating activity level. In winter, extreme environmental conditions in the sub-summit zone make it impossible to maintain heat equilibrium and lead to hypothermia. The emergency night in the sub-peak zone leads to gradual cooling of the body which in winter can cause severe hypothermia of the climber’s body. At altitudes < 7000 m, climbers should consider using clothing that allows variation of insulation and active regulation of their fit around the body.

**Supplementary Information:**

The online version contains supplementary material available at 10.1007/s00484-023-02594-1.

## Introduction

High-altitude mountaineering is becoming ever more popular (Burtscher 2004; Huey et al. 2020). More than 47,000 climbers have participated in expeditions to 8000 m peaks in the Himalayas (1950–2021), and about 40% of them have reached 8000 m summits (Salisbury and Hawley 2022). This number is boosted by the popularity of commercial expeditions, which were responsible for almost 75% of attempts in 2006 (Salisbury and Hawley 2007). For the two most popular commercial climbing routes on Mount Everest (Nepal: S Col-SE Ridge; Tibet: N Col-NE Ridge), the number of climbers attempting an ascent has risen by 60% over the past 15 seasons (Huey et al. 2020). Commercial expeditions to 8000 m peaks in the winter season are also gaining popularity (Benavides 2021).

Mountaineers, especially in the Himalayas, must face harsh weather conditions that can be compared to polar ones (Havenith 2010; Szymczak and Błażejczyk 2021). These conditions are characterized by low air temperature (Ta) and high wind speed (v) (Szymczak et al. 2021a). Additional climate features specific to the high mountain environments are the air pressure (ap)—which results in reduced air density and oxygen content that decrease with altitude (Huey et al. 2001; Kandjov 1997) and the increasing intensity of solar radiation (due to low optical mass and high transparency of the atmosphere and a large share of radiation reflected from snowy slopes) (Błażejczyk et al. 2013; Szymczak and Błażejczyk 2021).

The main bulk of research dealing with the impacts of the Himalayan climate on human physiology focuses on ap, Ta, and v . Barometric pressure determines the partial pressure of inspired oxygen (P_i_O_2_), which in turn affects maximum oxygen uptake ($\dot{\mathrm{V}}{\mathrm{O}}_2\max$), and through this limits the speed of vertical ascent (Bailey 2001; Matthews et al. 2020a; West et al. 1983; West et al. 2007b; West and Wagner 1980). Low Ta and high v mainly determine the risk of hypothermia and frostbite (Ainslie and Reilly 2003; Huey and Eguskitza 2001; Araźny and Błażejczyk 2007; Szymczak et al. 2021a; Parsons 2003). To determine such risks, most studies use various simple biometeorological indicators, such as wind chill temperature (WCT) (Osczevski and Bluestein 2005) and facial frostbite time (FFT) (Moore and Semple 2011; Szymczak et al. 2021a, 2021b; Tikuisis and Osczevski 2003). These indicators only take into account Ta and v, while ignoring other physical features of the atmosphere important in the mountains, such as ap, solar radiation, or air density.

In maintaining the body’s heat equilibrium, the influence of atmospheric factors as well as physiological and behavioral factors, such as physical activity, thermal insulation, and thickness and color of clothing, are equally important. Experimental thermophysiological research carried out outdoors in alpine and arctic conditions is scant (Blażejczyk 1994; Błażejczyk et al. 2008, 2013; Błażejczyk and Kunert 2011; Błażejczyk and Twardosz 2002; Cena et al. 2003).

There are only a few studies describing the influence of the weather conditions on the body’s ability to maintain thermal balance in the Himalayas. Cena and Tapsell (2000) as well as Cena et al. (2003) examined the thermal comfort of student participants of a Himalayan expedition while staying in tents at different heights (2640–5170m). Szymczak et al. (2021b) investigated deaths >8000 m potentially associated with body cooling. Szymczak and Błażejczyk (2021) assessed how meteorological conditions at different altitudes affect heat transfer flows in individuals in different seasons while climbing Everest. They also examined how various wind speeds and clothing insulation levels influenced human heat balance.

The aim of the present research is to trace the heat exchange between humans and their surroundings during a typical, 6-day summit attempt of Everest in spring and winter. An additional case study of climbers spending a night outdoor, without tent protection, during a delayed descent from the peak is considered. We intend to make such simulations for actual, observed weather conditions and for typical clothing used by climbers in warm and cold seasons. The calculations focused on climbers who do not use supplemental oxygen and therefore do not wear oxygen masks.

## Materials and methods

### Materials

Daily variation of the heat balance components during Everest summit attempts were calculated in spring and winter seasons using meteorological data collected at automatic weather stations installed on the mountain (Nepal: S Col-SE Ridge) between 6464 m (Camp 2) and 8430 m (Balcony) during a National Geographic expedition (Matthews et al. 2020a, 2020b; National Geographic 2021). The data included Ta, air vapor pressure (vp), relative air humidity (RH), v, ap, global solar radiation (K_glob_), sky longwave radiation (La), and outgoing ground longwave radiation (Lg). The data represent the hourly values of the measured meteorological parameters—Supplementary Materials (SM) (Table SM1). The characterization of the meteorological data was done by Szymczak and Błażejczyk (2021).

The current study includes the measurement period from May 20, 2019, to January 6, 2020. The end date was chosen as v data are questionable after 6 January 2020 (Szymczak and Błażejczyk 2021). As typical in the Himalayan expeditions, the days of the summit attempt were chosen mainly because of the low v values (Peplow 2004). It turned out that most of spring peak attempts in 2019 took place between 20 and 25 May (225 successful ascents on the 22nd and 400 on the 23rd of May, Salisbury and Hawley 2022), and these days were chosen in our research. Summit attempts in the winter are rare, and in the analyzed season 2019/2020, none of the three expeditions to Everest undertook summit attempts. Nevertheless, in the studied period, weather conditions, mainly low v, indicate that the period favorable for such an attempt took place between December 21 and 26.

**Fig. 1:**
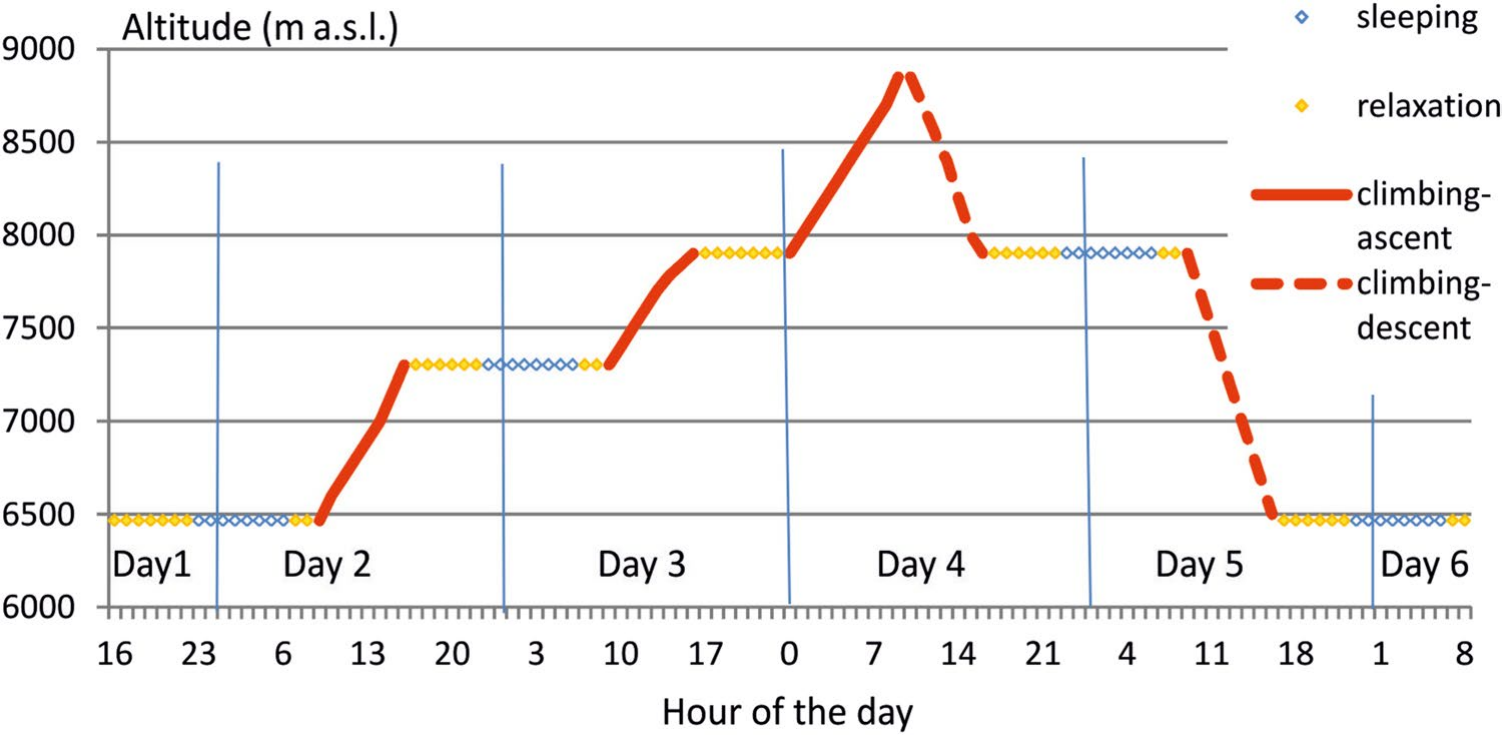
Phases of Mt. Everest summit attempts

In general, every day of activity in the Himalaya consists of four phases: morning relaxation in camp (6–8 a.m.), climbing (8 a.m.–4 p.m.), afternoon relaxation (4–10 p.m.), and sleeping (10 p.m.–6 a.m.). Our analysis of summit attempts covers 6 days. On the first day, the climbers spend the afternoon and night at Camp 2 (6464 m). On the second day, they climb up to Camp 3 (7300 m). There they spend the night, and during the third day, they climb to the South Col (7945 m). They rest there up to midnight when they start their final summit attempt. During the fourth day, they reach the summit at about 11 a.m. and next they go down to South Col, reaching it at about 4 p.m. After spending the night, they go down on the fifth day to Camp 2, reaching it at about 4 p.m. The analysis ends on the morning of the 6th day after spending the night in Camp 2 (Fig. 1).

### Methods

The components of the human heat balance were calculated with the use of an upgraded version of the Man-ENvironment heat Exchange_High Altitude model (MENEX_HA) (Blażejczyk 1994, 2005; Błażejczyk and Kunert 2011; Szymczak and Błażejczyk 2021). The model was upgraded to estimate dynamic changes of metabolic heat production, heat loss by conduction, and respiration at high altitude. The model is sensitive to changes in meteorological elements including air density and ap at different altitudes (Błażejczyk 2005).

The MENEX_HA calculates the basic components of the human heat balance in non-stationary conditions. The method of the calculations is described in the Annex. The general equation of heat transfer between humans and the environment is

1 $$\mathrm{M}+\mathrm{R}+\mathrm{L}+\mathrm{C}+\mathrm{E}+\mathrm{R}\mathrm{es}+\mathrm{C}\mathrm{d}=\mathrm{S}$$

The explanation of symbols and their units is provided in Table 1.

**Table 1:** List of symbols and units

ac	%	Albedo of skin and clothing
A_du_	m^2^	du Bois body surface area =1.9
ap	hPa	Air pressure
C	W⋅m^−2^	Heat exchange by convection
ca	n.d.	Contact area factor: 0.25 for sitting and 0.5 for laying person.
Cd	W⋅m^−2^	Heat exchange by conduction
cp	J*·*kg^−1^*·*K^−1^	Specific heat of air, assumed 1005
cp_v_	J*·*kg^−1^*·*K^−1^	Specific heat of water vapor, assumed 1996
d_a_	kg∙m^−3^	Absolute humidity of the inspired air
d_ex_	kg∙m^−3^	Absolute humidity of the expired air, assumed 2.9394
E	W⋅m^−2^	Heat loss by evaporation
f_cl_	n.d.	Dimensionless coefficient of the body covered by clothing (ISO 9920)
h	Degrees	Sun disk altitude
hc	W⋅m^−2^⋅K^−1^	Coefficient of heat transfer by convection
hc’	W⋅m^−2^⋅K^−1^	Coefficient of heat transfer by conduction within clothing
hdk	W·m^−2^·K^−1^	Dynamic coefficient of heat conduction through clothing, =0.055 (Kirucińska et al. 2016)
he	W⋅m^−2^⋅hPa^−1^	Coefficient of heat transfer by evaporation
h_fg_	J*·*kg^−1^*·*K^−1^	Enthalpy of vaporization
I_cl_	clo	Thermal insulation of clothing
Ie	n.d.	Coefficient reducing heat transfer through clothing (for E)
Irc	n.d.	Coefficient reducing heat transfer through clothing (for C, Cd, and R)
K_glob_	W⋅m^−2^	Global solar radiation
Kt	W⋅m^−2^	Potential solar irradiation at clear sky
L	W⋅m^−2^	Heat exchange by long wave (thermal) radiation
La	W⋅m^−2^	Thermal radiation emitted by sky (back radiation)
Lg	W⋅m^−2^	Thermal radiation emitted by ground
Ls	W⋅m^−2^	Thermal radiation emitted by human body
Lw	W⋅m^−2^	Thermal radiation emitted by tent’s walls and ground
M	W⋅m^−2^	Metabolic heat production
M_w_	W	Metabolic heat production
Mrt	°C	Mean radiant temperature
Ni	n.d.	Index of total cloud cover, = K_glob_/Kt
PiO_2_	hPa	Pressure of inspired oxygen
Q	W⋅m^−2^	Net radiation in man,
R	W⋅m^−2^	Absorbed solar radiation
RER	n.d.	Respiratory exchange ratio, assumed 0.85
Res	W⋅m^−2^	Heat loss by respiration
RH	%	Relative humidity of air
S	W⋅m^−2^	Net heat storage
s	n.d.	Absorbance/emittance coefficient, =0.97
SW	g∙h^−1^	Water loss from the body surface
Ta	°C	Air temperature
Ta_tent_	°C	Air temperature inside the tent
Tex	°C	Exhaled air temperature
Tsk	°C	Mean skin temperature
Tw	°C	Temperature of the particular tent’s walls and ground
v	m∙s^−1^	Wind speed
v’	m∙s^−1^	Velocity of subject movement, assumed 0.05
Vv	m^3^*·*s^−1^	Respiratory ventilation volume
Ve	l*·*min^−1^	Minute ventilation
VO_2_max	ml·kg^−1^·min^−1^	Maximal aerobic capacity for the specific altitude
vp	hPa	Actual air vapor pressure
vps	hPa	Vapor pressure at skin surface,
vps_max_	hPa	Saturated vapor pressure at the human body’s core temperature of 37°C, = 62.9
w	n.d.	Skin wettedness coefficient
σ	W⋅m^−2^⋅K^−4^	Stefan-Boltzmann constant, = 5,67 10^−8^
ρ	kg∙m^−3^	Density of the air

The model’s inputs include meteorological and physiological variables. The meteorological information it requires are Ta, v, vp, RH, ap, K_glob_, La, and Lg. The physiological data in the model include the climber’s sea level $\dot{\mathrm{V}}{\mathrm{O}}_2\max$, metabolic heat production (M), thermal insulation of clothing (I_cl_), clothing albedo, and speed of man movement. Some variables like mean skin temperature and skin wettedness are calculated using empirical formulas (see Annex).

#### Parameterization of meteorological variables

Meteorological variables were measured at the height of a standing person’s torso, i.e., about 1.2 m above ground level. Because of different forms of activity and various places of stay (outdoors/tent), the meteorological variables had to be parameterized in different ways. For climbing periods, we have applied meteorological variables measured at three stations, i.e., Camp 2, South Col, and Balcony.

During climbing (both, ascend and descend), the alpinists change their altitude. The altitudinal gradients of Ta, K_glob_, La, Lg, v, ap, and RH were calculated (between Camp 2 and South Col and between South Col and Balcony). The gradients were applied to define values of variables at each specific altitude on the track from Camp 2 to the top of Everest. Such gradients include also time changes between particular altitudes. To assess solar radiation at Balcony (it was not observed there), we use the altitudinal K_glob_ gradient estimated taking into account K_glob_ observed at Camp 2 and South Col. The gradient was used to extrapolate K_glob_ for all altitudes above South Col.

The values of meteorological parameters inside the tent were adapted. In the case of solar radiation, it was assumed that inside the tent, climbers were exposed only to diffuse radiation. In general, diffuse radiation consists of only 10–15% of K_glob_ under a clear sky and up to 40% in cloudy conditions (Błażejczyk 1997). Additionally, transfer of radiation is reduced by tent fabric. Thus, we have assumed that solar radiation intensity inside tents was 25 W·m^-2^ (in December and in early morning, i.e., 4–6 a.m. as well as in late afternoon, i.e., 7-8 p.m. in May; in these hours, K_glob_ observed outside tents was usually < 200 W·m^−2^) or 50 W·m^−2^ (in May, at 7–8 a.m. and 5–6 p.m.; the outside K_glob_ was 200–400 W·m^-2^). In the nighttime hours, K_glob_ is equal to 0 W·m^–2^.

The problem of air movement inside tents was not studied before. Thus, based on general knowledge of indoor climate, we arbitrarily used two v values inside tents: 0.3 m·s^−1^ for lower locations (Camp 2, Camp 3) where during the studied days outdoor wind was <4 m·s^−1^, and 0.7 m·s^−1^ for South Col where strong winds (>8 m·s^−1^) were noted.

In the case of RH, vp, and ap, it was assumed that inside the tents, their values were the same as outside.

No empirical information about in-tent temperature is available. For the sleep phase, it was assumed that the temperature in the tent is the same as outside. However, for the morning and afternoon relaxation periods, which are combined with the use of cookers to melt snow and to boil water, a special procedure was used to estimate Ta inside the tents. It was assumed that the tent has the shape of an equilateral cube, and the temperature of the four walls and the ground surface is equal to Ta. Due to the heat source usually placed under the tent ceiling, the temperature of the sixth wall (i.e., ceiling) was assumed to be 50°C. Such value refers to the physical fact that at an altitude of approximately 8000 m, the temperature of boiling water is only about 65°C, and considering the required long time to melt frozen snow and to boil water, we assumed a 50°C temperature of tent ceiling as a mean, constant value for whole relaxation period. Choosing this value, we also have took into consideration simulations made for ceiling temperature of 40 and 60°C. They influence heat flux changes within a small range. For example, the C value in the case of a ceiling temperature of 60°C is reduced of about 2 W·m^−2^, and the S value is higher of 3 W·m^−2^ in comparison to 50°C.

Using the Stefan-Boltzmann law, the amount of thermal energy emitted by walls and ground (Lw) was calculated for each wall according to the following formula:

2 $$\mathrm{Lw}=0.5\cdot\mathrm s\cdot\mathrm\sigma\cdot\left(273.13+\mathrm{Tw}\right)^4$$

Then, the average value of the heat energy intensity (Lw_avg_) was calculated, and next, its value was applied to calculate the air temperature inside the tent (Ta_tent_) according to the formula:

3 $${\mathrm{Ta}}_{\mathrm{tent}}={\left[\left({\mathrm{Lw}}_{\mathrm{avg}}\right)/\left(0.5\cdot \mathrm{s}\cdot \upsigma \right)\right]}^{0.25}\hbox{--} 273.13$$

#### Parameterization of physiological variables

Absorbed solar radiation is one of the sources of heat in humans outdoor. Its value depends on global (i.e., the sum of direct and diffuse radiation) and reflected fluxes (Błażejczyk et al. 1993). In mountain areas, K_glob_ observed on a horizontal surface depends not only on downward direct and diffused (by the atmosphere) radiation but also on diffused radiation (reflected from elevated parts of slopes). Thus, at the mountain slopes, K_glob_ has higher values than on flat locations (Błażejczyk 1998) as is the case for the Everest area. While the National Geographic database (National Geographic 2021) provides only K_glob_ values, we have used in our research the SolGlob model of absorbed solar radiation. The model was experimentally developed (Błażejczyk 2004) and is frequently applied in research based on K_glob_ data (Szymczak and Błażejczyk 2021).

The key physiological parameters determining heat balance in alpine climbing conditions are M (Ainsworth et al. 2011) and I_cl_ (Havenith 2010). Based on the experience of climbers in the Himalayas, we assumed that typical metabolism for the sleep phase is approximately equal to the basic metabolic rate, i.e., 60 W·m^−2^, and for the relaxation phases in the camp, M is equal to very light activity value, i.e., 75 W·m^−2^. For the climbing phase, metabolic rate was derived from maximal aerobic capacity values for the specific altitude ($\dot{\mathrm{V}}{\mathrm{O}}_2\max$). The latter was derived from sea level $\dot{\mathrm{V}}{\mathrm{O}}_2\max$ corrected for the impact of lower oxygen levels at altitude (Matthews et al. 2020a). M is then calculated for a climber with a body mass of 80 kg, body height of 1.7m, and body area of 1.9 m^2^ and for $\dot{\mathrm{V}}{\mathrm{O}}_2\max$ at a specific altitude (description of M parametrization is done in Annex).

Metabolic rate at maximal aerobic capacity (100% $\dot{\mathrm{V}}{\mathrm{O}}_2\max$) can only be sustained for short periods of climbing. Climbing takes place at submaximal levels, suggested to be between 50% (West et al. 2007a), 62% (55–72%) (Burtscher 2004) up to 85% (Bailey 2001) of the altitude specific $\dot{\mathrm{V}}{\mathrm{O}}_2\max$. As there is no clear agreement about the preferred rate of climbing, we have calculated the M value and corresponding human heat balance for three levels of activity, namely, 50%, 60%, and 70% of $\dot{\mathrm{V}}{\mathrm{O}}_2\max$.

The key to maintain a relative thermal equilibrium in the Himalayan weather conditions is the use of appropriate clothing in each of the phases of daily activity. I_cl_ used when climbing in the Himalayas was adopted on the basis of ISO 11079 (2007), ISO 9920 (2007), Havenith’s research (2010), and research conducted during the creation of the Universal Thermal Climate Index (UTCI) (Havenith et al. 2012). Information on clothes used by mountaineers during expeditions to 8000 m peaks allowed for I_cl_ assessment (Table SM2).

It was assumed that I_cl_ during climbing was, depending on altitude, 4.5–5.5 clo in spring and 5.5–6.0 clo in winter. The phase of relaxation needs less insulated clothing due to reduced v and elevated Ta in tents. However, during the sleeping phase, alpinists use both, clothing and cover materials (sleeping bags), which provide better insulation. To assess I_cl_ in those phases, the empirical equation proposed by McCullough and Kim (1996) was used. It estimates I_cl_ based on the thickness (mm) and number of clothing layers covering arms and calves as follows:

4 $${\mathrm{I}}_{\mathrm{cl}}=0.017\cdot {\mathrm{C}}_{\mathrm{th}\_}\mathrm{a}+0.101\cdot {\mathrm{C}}_{\mathrm{th}\_}\mathrm{c}+0.212\cdot {\mathrm{C}}_{\mathrm{la}\_}\mathrm{a}+0.347\cdot {\mathrm{C}}_{\mathrm{la}\_}\mathrm{c}+0.317$$

where C_th__a is clothing thickness on the arm, C_th__c is clothing thickness on the calf, C_la__a is the number of layers on the arm, and C_la__c is the number of layers on the calf.

One should note that I_cl_ values in Table SM2 refer to static conditions and do not consider reduction related to wind. However, to calculate coefficients reducing heat transfer through clothing (Irc, Ie), the effective insulation (Ief, according to Fourt and Hollies 1970) influenced by wind and body movement is applied:

5 $$\mathrm{Ief}=\mathrm{Icl}\cdot\left[1-0.27\cdot\left(\mathrm v+\mathrm v'\right)^{0.4}\right]$$

## Results

### Meteorological conditions during summit ascent days

Figure SM1 shows the course of the meteorological parameters during summit attempts (spring: 20–25th May; winter 21–26th December). Barometric pressure and Ta were steadily decreasing with altitude and were lower in winter than in spring at each stage of the summit ascent. Barometric pressure was on average 11 hPa lower in winter than in spring, and on the summit in winter, ap reached only about 330 hPa. Air temperature was also significantly lower in December than in May, and at the culmination of summit attempts in winter, Ta was about −40°C. The values of K_glob_ were similar during all stages of summit ascents with higher values during spring. Days during both attempts were sunny, and the amount of solar radiation depended on seasonal sun altitude and day length.

Wind speed was the most unstable parameter especially during the spring summit ascent. At the end of May, v changed from about 1 m·s^−1^ in Camp 2 up to 11 m·s^−1^ at the summit. When descending from the South Col, a 3-h episode of strong wind, up to 15 m·s^−1^, was also noted. In December, v fluctuated from 4–6 m·s^−1^ in lower locations to 12–14 m·s^−1^ in the sub-summit area. Water content in air (represented by vp) is relatively small and varied from 0.3 to 3 hPa in May and from 0.1 to 1 hPa in December. However, RH changed significantly, from 18 to 85% in May and from 20 to 80% in December.

### The daily cycle of the human heat balance

The meteorological conditions had a significant impact on the human heat balance during various phases of activity during the summit attempts, and they differ between spring and winter. The R, C, Cd, and L fluxes are primarily dependent on meteorological parameters; therefore, their intensity varies between May and December especially during climbing.

The external source of heat for the body is the absorbed solar radiation (R). Despite the high intensity of K_glob_ (especially in May, Fig. SM1), the thick clothing barrier causes that only a small part of the incoming radiation can be absorbed by the body surface. There are seasonal differences in R. Its value during climbing in May is about two times higher than in December. During daylight hours, the R flux changes in May from about 15 W·m^−2^ in the lower part of the route to about 20 W·m^−2^ in the peak parts. In December, the R values were 10 and 12 W·m^−2^, respectively. While staying in the tent, the R flux has negligible values. The amount of R only to a small extent compensated for heat loss (Fig. SM2).

The greatest daily and seasonal differences are observed in the case of convective heat losses (C). The intensity of C strongly depends on Ta and v, which clearly change with elevation, i.e., decrease in Ta and increase in v (Fig. SM1). While on the lower section of the route (day 2), the values of C did not exceed −35 W·m^−2^ in May and −40 W·m^−2^ in December, in the peak parts (day 4), the C was −50 and −90 W·m^−2^, respectively. The highest convective heat losses of −82 W·m^−2^ (May) and −93 W·m^−2^ (December) took place on the 5th day during the descent from the South Col camp and were related to a short episode of v increase (Fig. SM1). In the sleep and relaxation phase in the tent where air movement was limited and Ta was a few degrees Celsius higher than outside the tent, the average values of C range from −16 W·m^−2^ in December to −13 W·m^−2^ in May (Fig. SM2, Table 2).

**Table 2:** Average (Avg), maximum (Max), and minimum (Min) values of R, C, Cd, L, and of M, E, Res, and S fluxes (W·m^−2^) at different levels of physical activity (−50, −60, −70% of $\dot{\mathrm{V}}{\mathrm{O}}_2\max$) during summit attempts in May and December 2019

Heat flux	May					December				
	Max (whole period)	Min (whole period)	Avg (whole period)	Avg (climbing)	Avg (sleep, relax)	Max (whole period)	Min (whole period)	Avg (whole period)	Avg (climbing)	Avg (sleep, relax)
R	27.4	0.0	6.1	13.6	0.6	12.3	0.0	2.3	5.9	0.1
C	−10.1	−81.6	−23.5	−37.3	−12.9	−12.5	−93.2	−34.9	−63.9	−16.3
Cd	0.0	−13.3	−4.0	0.0	−6.3	0.0	−19.3	−5.6	0.0	−9.0
L	−13.8	−27.4	−19.6	−15.7	−21.6	−13.8	−30.3	−21.1	−16.9	−23.7
M-50	206.4	60.0	103.9	161.0	68.2	202.5	60.0	101.6	152.1	68.1
M-60	247.7	60.0	116.2	193.2	68.2	243.0	60.0	113.5	182.5	68.1
M-70	289.0	60.0	128.4	225.4	68.2	283.4	60.0	125.4	212.9	68.1
Res-50	−2.4	−48.4	−18.7	−38.0	−9.2	−2.3	−92.1	−19.1	−43.1	−6.1
Res-60	−2.4	−60.8	−22.1	−47.6	−9.2	−2.8	−110.6	−22.9	−51.8	−7.3
Res-70	−2.4	−73.2	−25.4	−57.2	−9.2	−3.2	−129.0	−26.9	−60.7	−8.5
E-50	0.0	−57.3	−15.4	−38.2	−1.2	0.0	−56.7	−14.8	−34.8	−1.5
E-60	0.0	−74.6	−20.6	−51.7	−1.2	0.0	−73.7	−19.8	−47.6	−1.5
E-70	0.0	−92.0	−25.7	−65.3	−1.2	0.0	−90.8	−24.8	−60.3	−1.5
S-50	110.9	−3.5	29.2	45.2	18.3	80.5	−76.0	7.4	−2.8	11.3
S-60	130.7	−3.5	32.9	54.3	18.3	99.1	−79.4	11.1	6.1	11.3
S-70	150.4	−3.5	36.7	63.3	18.3	117.8	−82.7	14.8	14.7	11.3

Source: own derivation

The intensity of the long wave heat loss flux (L) is similar in both seasons and ranges from about −23 W·m^−2^ during relaxation and sleep to about −17 W·m^−2^ during climbing. Only occasionally L flux exceeded the level of −30 W·m^−2^. Similar values are also assumed by the flux of conductive heat losses (Cd), which occur only in the sleep and relaxation phases. The average Cd values range from −6 (in May) to −9 W·m^−2^ (in December). The highest intensity of this flux does not exceed −20 W·m^−2^ (Fig. SM2).

A key element of the human heat balance is the amount of heat generated in metabolic processes. While constant values were assumed for the sleep and relaxation phases (60 and 75 W·m^−2^, respectively), then for the climbing phases, M depended on P_i_O_2_, $\dot{\mathrm{V}}{\mathrm{O}}_2\max$—which differs according to altitude and on the level of activity (50, 60, and 70% of $\dot{\mathrm{V}}{\mathrm{O}}_2\max$).

Metabolism values determined in this way affect the size of those heat fluxes that depend on the value of metabolism, i.e., the heat loss due to evaporation from the body surface (E) and heat loss through respiration (Res), and consequently also the net heat storage (S). Metabolic values were similar in both seasons. In the peak zone, M values during the climbing were significantly lower than in the lower part of the route, reflecting the reduced $\dot{\mathrm{V}}{\mathrm{O}}_2\max$. At 50% $\dot{\mathrm{V}}{\mathrm{O}}_2\max$, M varied from approximately 200 W·m^−2^ at the bottom of the trail to 140 W·m^−2^ (spring) and 115 W·m^−2^ (winter) at the summit of Everest. At the level of 60% $\dot{\mathrm{V}}{\mathrm{O}}_2\max$, M changed from 250 to 150−130 W·m^−2^, respectively, and at 70% $\dot{\mathrm{V}}{\mathrm{O}}_2\max$, from 280 to 180–150 W·m^−2^ (Fig. SM3, Table 2).

The level of metabolism, combined with changing meteorological conditions (Ta, vp, v) clearly differentiated the amount of E and Res fluxes (Fig. SM4). In the case of E flux, when both seasons are considered, its intensity >8000 m varied from −20 to −30 W·m^−2^ at 50% $\dot{\mathrm{V}}{\mathrm{O}}_2\max$ and from −40 to −60 W·m^−2^ at 70% $\dot{\mathrm{V}}{\mathrm{O}}_2\max$. Heat losses by respiration, on the other hand, are the most intense when climbing in the top part of the route (to −70 W·m^−2^ in May and to −121 W·m^−2^ in December). This was due not only to the low content of water vapor in the air and its low temperature and density, but also to extreme hyperventilation >8000 m.

The result of heat gains (M and R fluxes) and losses (fluxes: C, E, L, Res, and Cd) is the balance of its exchange, i.e., net heat storage (S) (positive or negative). In May, the total range of the S flux varied from −3.5 to +150.4 W·m^−2^. In December, the range of S variability ranged from −76.0 to nearly +118 W·m^−2^ (Table 2).

**Fig. 2:**
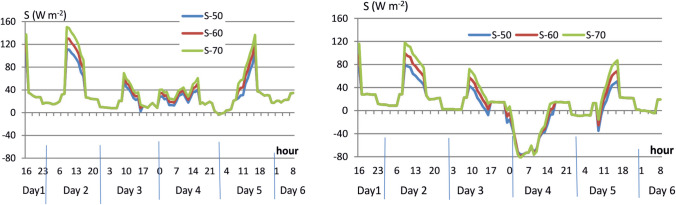
Changes in net heat storage (S) during summit attempts in May (left panel) and in December (right panel) 2019. Source: own derivation

There is a clear daily cycle of the S flux. During the relaxation and sleep phases, the S values range in spring from 0 to +26 W·m^−2^. In winter, during these phases, S drops below 0, especially in high camps (South Col, Camp 3). When climbing, in the lower parts of the route (between Camp 2 and Camp 3), the net heat storage is up to +150 W·m^−2^ in May and up to +120 W·m^−2^ in December. In the top parts of the route, the heat exchange balance oscillates around +20–40 W·m^−2^ in May. In December, >8000 m S has negative values (from −50 to −80 W·m^−2^). This is primarily the result of very large convective heat losses (caused by very low Ta and high v) and respiration (due to increased ventilation at this altitude). Such negative S during winter summit attempt would lead to hypothermia (Fig. 2).

### Emergency night

**Fig. 3:**
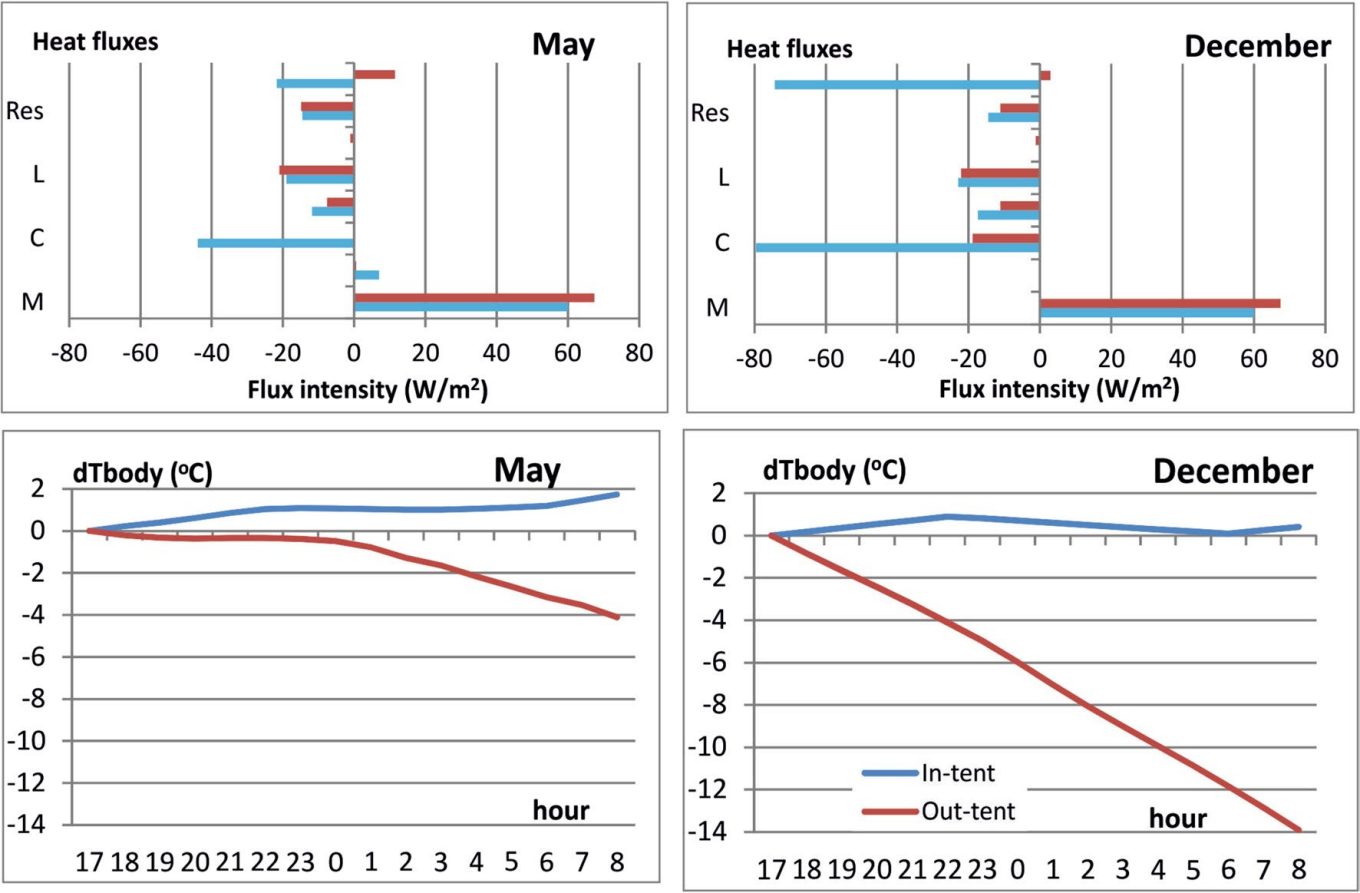
Mean values of particular heat balance fluxes (upper panel) and changes in body temperature (dTbody) during nights spent outside (out-tent) and inside (In-tent) tent in May and in December (lower panel)) 2019. Source: own derivation

In case of unforeseen circumstances that prevent a return to the South Col, mountaineers must spend the night without a tent, sleeping bags, and warm drinks. They are exposed to the low temperature prevailing at 8000 m and to strong winds, which intensify convective heat loss. In May, during the night spent outside the tent, C values are almost three times higher, and in December, more than four times higher than during the night in the tent (Fig. 3). An additional factor that increases C is the lower I_cl_ than in a tent (no sleeping bag). The values of the remaining heat exchange fluxes were similar to those simulated for in-tent night.

The consequence of high C values is a lower net heat storage in climbers without a tent. While the average values of S in the tent were close to zero (in May +11.5 W·m^−2^, in December +3.0 W·m^−2^), then outside the tent, they were respectively −21.8 and −74.4 W·m^−2^. In May, from midnight, the body cooling process intensified as a result of a decrease in Ta and an increase in v. In December, S had negative values throughout the whole night (from −70 to −90 W·m^−2^).

The changes in body heat content were on average about −78 kJ·m^−2^·h^−1^ in May. In December, these losses reached as much as −268 kJ·m^−2^·h^−1^. For in-tent night, such changes in body heat content were 41.3 and 10.8 kJ·m^−2^·h^−1^, respectively. According to Smolander (1987) and Davis (2020), a change of body heat content of 290 kJ leads to a rise/fall of body temperature of 1°C. Spending the night out of a tent in May led to a decrease of body temperature of about 4°C. In December, the body temperature would decrease dramatically by about 14°C, which would lead to severe hypothermia and death (Fig. 3).

## Discussion

### Choosing the optimal weather window

Improved weather forecasting was one of the reasons for the higher chance of summiting Everest and the lower risk of death (Huey et al. 2020). Summit attempts are planned if favorable wind conditions are forecasted at least for the day of ascent and descent from the summit (Peplow 2004). However, weather parameters that mostly determine the risk of hypothermia at high altitude include not only wind but also ap and Ta (Szymczak and Błażejczyk 2021). Additionally, the speed of climbing and the risk of acute altitude illness (AAI) are determined by the level of ap (Matthews et al. 2020a). Therefore, the weather forecast should optimally include v, ap, and Ta.

Our results show that mountaineers who are limited to choosing the summit attempt period based on the wind forecast alone should optimally choose days with winds <10–15 m·s^−1^ and <5 m·s^−1^ for spring and winter ascents, respectively. Due to the rarity of low wind conditions on Everest in winter, winter ascents without oxygen carry an extreme risk of severe hypothermia and death and are almost impossible to perform.

Matthews et al. (2020a) suggested that forecasts of oxygen availability (through ap) should be standard during no-oxygen ascents. Moore and Semple (2011) concluded that ap could act as a predictor of hypothermia and frostbite. We strongly support the above suggestions. The value of ap might be difficult to interpret by climbers; therefore, presenting it in terms of perceived altitude should be considered (Matthews et al. 2020a; Szymczak et al. 2021a).

The indicators for the risk of hypothermia or frostbite include WCT, FFT, and UTCI (Błażejczyk et al. 2012). Unfortunately, they consider the sea-level air density and they apply at high altitude is fraught with error. By contrast, the MENEX_HA model considers air density and ap at high altitude and provides a net value of heat balance which was applied to assess functional and survival time as indicators of hypothermia risk according to Keefe and Tikuisis (2008).

### Cold exposure survival time

Our methodology of calculating the partitional calorimetry of the human heat balance at high altitude may be the base for the functional and survival time prediction of hypothermic stress at extreme altitudes. Based on an average specific heat of body tissue of 3.48 J·g^−1^·K^−1^, Smolander (1987) and Davis (2020) suggest that the change in body heat equal to 290 kJ leads to an increase or drop in body temperature by 1°C. In order to estimate cold exposure survival of drowning victims, Keefe and Tikuisis (2008) used two parameters: functional time, i.e., period when a cognitive self-help is possible (between 37 and 34°C of body temperature) and survival time which ends when the body temperature drops <28°C.

According to our results, the clothing insulation of 5.5 clo and activity level of 50% $\dot{\mathrm{V}}{\mathrm{O}}_2\max$ are enough to keep positive net heat storage (+20 to +40 W·m^−2^) during summit attempt >8000 m in May in favorable weather conditions (Ta=−20°C, v<11 m·s^−1^). Such conditions are close to the monthly average of May on the Everest summit (Ta=−26±1°C, v=16±3 m·s^−1^) (Szymczak et al. 2021a) and those present during 210 no-oxygen Everest ascents (Ta=−25±3°C, v=12±5 m·s^−1^) (Szymczak et al. 2021b). Similarly to our results, Havenith (2010) found that a climber without supplemental oxygen at the Everest’s summit in non-winter conditions (Ta=−25°C, v=11 m·s^–1^) needs clothing insulation of 4.5 clo to keep thermal balance.

On the other hand, despite clothing insulation of 6.0 clo and activity level between 50 and 70% $\dot{\mathrm{V}}{\mathrm{O}}_2\max$, weather conditions >8000m in December (Ta of −35/−40°C, v 12–14 m·s^−1^) cause negative net heat storage (−50 to −80 W·m^−2^ or −180 to −288 kJ·m^−2^·h^−1^). According to Havenith’s calculations in Ta=−40°C and v=11 m·s^–1^, a climber should have insulation of 6.5 clo (Havenith 2010). In fact, wind reduces the effective insulation of clothing (Fourt and Hollies 1970) which can be lower than those estimated for static conditions. This can lead to the negative values of net heat storage observed during climbing >8000 m in December. The loss of heat (−180 to −288 kJ·m^−2^·h^−1^) causes a 0.6 to 1°C drop in body temperature per hour (Davis 2020; Smolander 1987). It means a high risk of moderate to severe hypothermia during the summit day and very low chances for successful ascent. It has to be underscored that the average v on the summit of Everest in midwinter is significantly higher (41 m·s^−1^) than during the chosen weather window (Szymczak et al. 2021a). So far, there was only one winter no-oxygen ascent of Everest with Ta=−33°C and v=26 m·s−^1^ on the summit and 14 ascents with oxygen (average Ta=−36°C, average v=36 m·s−^1^) (Szymczak et al. 2021b). The only winter no-oxygen ascent of K2 (8611 m) was in an exceptionally favorable weather window with Ta of −35 to −40°C and v of 5–9 m·s^−1^ on the summit (Matthews et al. 2021; Szymczak et al. 2021b).

In the analysis of heat balance during a night without a tent, we found that the approximate rate of decrease in body temperature is about 1°C every 3.5 h in spring and 1°C every 1 h in winter. Therefore, functional time in spring ends after 10.5 h and in winter only after 3 h of exposure. Survival time in spring lasts 31.5 h, whereas in winter, it would only be 9 h, which suggests that surviving a night in winter without shelter is very unlikely. The cases of two climbers who had to bivouac >8500 m presented by Moore and Semple (2012) seem to confirm the above calculations and assumptions. The climber who died was exposed to conditions similar to our winter attempt (Ta=−31°C, v=15 m·s^−1^), and the one who survived experienced typical spring conditions (Ta=−23°C, v=2 m·s^−1^). The calculated survival times under sedentary conditions done by Tikuisis (1995) have the same order of magnitude as ours: 18 h (Ta −20°C and v 14 m·s^–1^) and 6 h (Ta −40°C and v 14 m·s^–1^) (Tikuisis 1995). Thus, high negative S in the summit zone in winter leads to hypothermia both during climbing and spending the night without shelter. It should perhaps be considered that the survival time based on the physiological state with water immersion, where a hypothermic person is found and then rescued, is perhaps not relevant close to the summit of Everest, as there, the rescue of an immobilized person with a body temperature between 34 and 28°C is virtually impossible.

The issue that should be signaled in the discussion is the effect of heat generated in the shivering process on changes in body temperature. This process is initiated when the core temperature decreases to 35°C and is significantly reduced to <31°C (Haman et al. 2007; Haman and Blondin 2017). Heat production through shivering can reach values equivalent to five times resting M or 40% of $\dot{\mathrm{V}}{\mathrm{O}}_2\max$ (Eyolfson et al. 2001). Shivering affects the heat balance calculation, reduces negative storage, and lengthens the time to reach 290 kJ. However, its effectiveness and role in extreme hypoxia conditions need to be explored.

### Non-traumatic causes of death at extreme altitudes

Firth et al. (2008) observed that severe weather is the main factor responsible for about 25% of fatalities >7000m on Everest. The authors pointed out that hypothermia, along with AAI, was the leading cause of non-traumatic deaths. However, out of 94 mountaineers who died >8000 m, only 6 had symptoms indicative of hypothermia. Our results show that the risk of hypothermia during the simulated exposures (no excessive weather conditions) mainly concerns those mountaineers who are forced to bivouac without a shelter or climb >8000 m in the winter season. The rarity of such bivouacs and winter climbs might explain the low hypothermia death toll observed by Firth et al. (2008). Thus, hypothermia primarily threatens the lives of climbers during non-shelter bivouac accompanied by extreme weather conditions, while hypoxia is the main cause of deterioration for mountaineers in the active phase of climbing, those staying in a tent in extreme weather conditions or without shelter in favorable weather conditions (Moore and Semple 2012; Sawicka and Szymczak 2023).

### Clothing insulation

In our study, the amount and type of clothing worn were taken from practical experiences of Himalaya’s climbers. Adding or taking away clothing would affect its insulation and result in different values of heat balance. In fact, the high metabolic rate values during climbing phases together with high values of clothing insulation lead to the accumulation of heat in the body. High positive net heat storage values (between 120 and 150 W·m^−2^) calculated for the phase of climbing in lower parts of the summit attempt suggest that I_cl_ of 4.5 clo in spring and 5.5 clo in winter when climbing at 50–70% $\dot{\mathrm{V}}{\mathrm{O}}_2\max$ may cause a significant rise in body temperature and hyperthermia. According to Smolander (1987) and Davis (2020), 150 W·m^−2^, i.e., 540 kJ·m^−2^·h^−1^, would cause almost 2°C increase in body temperature per hour. It would be expected that climbers reduce the amount of clothing worn when they feel warm and thereby limit heat accumulation. For UV protection, however, a minimum skin coverage is required. Further, the clothing insulation values were not reduced for the impact of movement and wind (Havenith and Nilsson 2004). Without these reductions and the behavioral clothing reductions in the model, the positive heat storage observed may be considered a worst case (Chen et al. 2004; Havenith et al. 1990).

### Characteristics of partitional calorimetry at high altitude environment

The most important feature of a high altitude environment is a drop of air density with altitude which is an effect of a decrease in Ta and ap. Lower density provides better insulation in the near-body air layer, which reduces convective heat loss (Kandjov 1997). Extremely low ap at altitudes >8000 m reduces convective heat loss by almost 50% compared with altitudes <4000 m in the same temperature (Huey et al. 2001; Szymczak and Błażejczyk 2021). Therefore, the formula for calculating convective heat loss at high altitude must include ap as we did in our study (see Annex).

As with an increase of altitude, the same level of exertion requires higher minute ventilation due to the lower P_i_O_2_; the typical formulas for calculating Res at sea level such as ISO 7933 (1989) underestimate its value. The formula for calculating Res at high altitude should contain both air density and parameters of minute ventilation as it is in the method proposed by Cain et al. (1999) used in our study (see Annex). Our results show that changes in minute ventilation have an important role in assessing Res at high altitude. In winter, during climbing in the sub-peak zone, the Res flux has the biggest impact in heat loss in climbers.

### Limitations

M values during the climbing were derived from $\dot{\mathrm{V}}{\mathrm{O}}_2\max$ representative of very fit, male mountaineers corrected for the altitude effect, with sea level $\dot{\mathrm{V}}{\mathrm{O}}_2\max$ close to 60 ml·kg^−1^·min^−1^. Given the range of submaximal $\dot{\mathrm{V}}{\mathrm{O}}_2$ values reported in the literature, heat transfer balance and its components (M, E, Res) were calculated for 3 rates of climbing: 50, 60, and 70% $\dot{\mathrm{V}}{\mathrm{O}}_2\max$. Considering the variability in individuals’ aerobic capacity and preferred rate of climbing, our results may be either underestimated (for climbers with higher $\dot{\mathrm{V}}{\mathrm{O}}_2\max$ and rate of climbing) or overestimated (for climbers with lower $\dot{\mathrm{V}}{\mathrm{O}}_2\max$ and rate of climbing). Our study assumed that climbing in the sub-peak zone was done without the use of oxygen apparatus. Therefore, our simulations refer to this group of climbers. For climbing with oxygen support, the obtained values of metabolism, but also of respiratory heat loss, may be different from those obtained in the present research. The fact that the most extreme weather conditions on the summit of Everest (Ta=−36±4°C, v=36±7 m·s^−1^) were survived during 14 winter ascents with oxygen support may prove its important role in keeping the net heat storage positive (Szymczak et al. 2021b). The effect of oxygen support on the heat balance at extreme altitudes remains to be determined.

The heat balance analysis was conducted only for one climbing weather window in each season. Climbing in worse weather conditions or during weather deterioration would result in lower values of net heat storage, as mentioned by Szymczak and Błażejczyk (2021) with increasing hypothermia risk. The simulation of the heat balance during Everest attempts in spring and winter can be considered representative of other 8000 m Himalayan peaks at given altitudes, but should not be transposed to the Karakoram. Due to the more northern location of Karakoram, Ta and ap in the winter season are significantly lower at given altitudes than in the Himalayas (Szymczak et al. 2021a).

Due to the lack of observational data, we assumed in our simulations some simplifications of meteorological data that can be noted inside tents (temperature, air movement, solar radiation). This point needs future experimental research in the Himalayas environment to find the most adequate values of such variables.

## Conclusions

In spite of extreme environmental conditions in the sub-summit zone of Everest, it is possible to keep heat equilibrium of the climbers’ body during spring summit attempts. This can be achieved by the use of appropriate clothing (as applied in our research) and by regulating activity level. In spring attempts, when heat exchange balance has relatively high positive values (especially at 70% $\dot{\mathrm{V}}{\mathrm{O}}_2\max$), climbers should consider using less-insulative clothing or clothing that allows variation of insulation and active regulation of their fit around the body. A relative surplus of heat, which occurs during the climbing phase, can be reduced during the relaxation and sleeping phases.

In winter, extreme environmental conditions in the sub-summit zone make it impossible to maintain heat equilibrium and lead to hypothermia. Reduction of heat loss by convection (development of more insulative clothing assembles) and reduction of heat loss by respiration (research on face masks or materials) might enable future winter ascents.

The emergency night in the sub-peak zone without a tent leads to gradual cooling of the body. While in the spring season, it does not lead to fatal body cooling, in winter, it is so large that it can cause severe hypothermia. The emergency summit kit of every mountaineer climbing >8000m should include an emergency shelter and rescue bag. The important role of limiting wind exposure by sheltering in the snow cover or snow cave during bivouac without a tent should be emphasized.

### Supplementary Information

ESM 1 (DOCX 890 kb)

## Annex

Equations used in the Man ENvironment heat Exchange-High Altitude model (MENEX_HA)

### Metabolic heat production (M)

It was assumed that typical metabolism (M) for the sleep phase is approximately equal to the basic metabolic rate, i.e., 60 W·m^−2^, and for the relaxation and morning phases in the camp, M is equal to very light activity value, i.e., 75 W·m^−2^. However, for the climbing phase, the metabolic rate was derived from maximal aerobic capacity ($\dot{\mathrm{V}}{\mathrm{O}}_2\max$).

The calculations were based on data published by other authors and are representative for well-trained climbers, with a sea level $\dot{\mathrm{V}}{\mathrm{O}}_2\max$ of approximately 57 ml·kg^−1^·min^−1^ (Matthews et al. 2020a; Bailey 2001; Pugh 1962; Sutton et al. 1988; West et al. 1983).

First, the air pressure (ap) was converted to VO_2_max by calculating the partial pressure of inspired oxygen (PiO_2_):

6 $${\mathrm{PiO}}_2=0.2095\cdot \left(\mathrm{ap}-{\mathrm{vps}}_{\mathrm{max}}\right)$$

where 0.2095 is the volume fraction of oxygen in the atmosphere (Wallace and Hobbs 2006).

Next, following the calculations of Matthews et al. (2020a), we used a rearranged regression equation of Bailey (2001) to obtain $\dot{\mathrm{V}}{\mathrm{O}}_2\max$ of acclimatized individuals as a function of PiO2:

7 $$\dot{\mathrm{V}}{\mathrm{O}}_2\max =\left[\ln \left({\mathrm{PiO}}_2\cdot 0.75\right)-3.25\right]/0.0308$$

In the following step, the $\dot{\mathrm{V}}{\mathrm{O}}_2\max$ was recalculated to metabolic rate (M_w_) using an equation proposed by Cramer and Jay (2019):

8 $$\begin{aligned}{\mathrm{M}}_{\mathrm{w}}&=\dot{\mathrm{V}}{\mathrm{O}}_2\max\ \left\{\left[\left(\left(\mathrm{RER}-0.7\right)/0.3\right)\cdot 21.13\right]\right.\\&+\left.\left[\left(\left(1.0-\mathrm{RER}\right)/0.3\right)\cdot 19.62\right]\right\}\cdot 1000/60\end{aligned}$$

Basing on the meta-analysis of Griffiths et al. (2019) and suggestions of Cramer and Jay (2019), it was assumed the RER value during high-altitude climbing to be equal to 0.85, which is typical for low to moderate work intensities. Thus, Eq. (3) can be simplified to

$$\mathrm{Mw}=\dot{\mathrm{V}}{\mathrm{O}}_2\max \cdot 339.6$$

When including in Eq. (3) $\dot{\mathrm{V}}{\mathrm{O}}_2$ (represented 50, 60 or 70% of $\dot{\mathrm{V}}{\mathrm{O}}_2\max$), for the alpinist with 80 kg of body weight, 1.9 m^2^ of du Bois body area, and 1.77 m of body height, the following equation is received:

9 $$\mathrm{M}=\dot{\mathrm{V}}{\mathrm{O}}_2\left(339.6/80\cdot 1.77\cdot 1.9\right)$$

or

10 $$\mathrm{M}=\dot{\mathrm{V}}{\mathrm{O}}_2\cdot 14.3$$

### Radiation balance (Q)

Radiation balance (Q) is the sum of absorbed solar radiation (R) and net long-wave (i.e., thermal) radiation (L):

11 $$\mathrm{Q}=\mathrm{R}+\mathrm{L}$$

The SolGlob model developed by Błażejczyk (1998, 2004) based on empirical research was used to calculate the absorbed solar radiation. Given the information on the intensity of global solar radiation (K_glob_), the formulas for calculating the R value have various forms, depending on the height of the sun (h) and an index of total cloud cover (Ni). The Ni is a fraction of the actual Kglob in the theoretically possible value of global radiation in a cloudless sky (Kt, Ni = Kglob/Kt), where Kt is calculated as follows:

12 $$\mathrm{Kt}=-0.0015\cdot {\mathrm{h}}^3+0.1796\cdot {\mathrm{h}}^2+9.6375\cdot \mathrm{h}-11.9$$

The formulas for calculating the *absorbed solar radiation* (*R*) have the following form:

- for h ≤ 12:

13 $$\mathrm R=\left(0.0014\cdot{\mathrm K_{\mathrm{glob}}}^2+0.476\cdot\mathrm{Kglob}-3.8\right)\cdot\left(1-0.01\cdot\mathrm{ac}\right)\cdot\mathrm{Irc}$$

- for h > 12° and Ni ≤ 0.8:

14 $$\mathrm{R}=0.247\cdot {{\mathrm{K}}_{\mathrm{glob}}}^{0.9763}\cdot \left(1-0.01\cdot \mathrm{ac}\right)\cdot \mathrm{Irc}$$

- for h > 12° and Ni from 0.81 to 1.05:

15 $$\mathrm{R}=3.692\cdot {{\mathrm{K}}_{\mathrm{glob}}}^{0.5842}\cdot \left(1-0.01\cdot \mathrm{ac}\right)\cdot \mathrm{Irc}$$

- for h > 12° and Ni from 1.06 to 1.2:

16 $$\mathrm{R}=43.426\cdot {{\mathrm{K}}_{\mathrm{glob}}}^{0.2326}\cdot \left(1-0.01\cdot \mathrm{ac}\right)\cdot \mathrm{Irc}$$

- for h > 12° and Ni > 1.2:
  17 $$\mathrm R=8.928\cdot{{\mathrm K}_{\mathrm{glob}}}^{0.4861}\cdot\left(1-0.01\cdot\mathrm{ac}\right)\cdot\mathrm{lrc}.$$

In the equations above, particular variables are calculated as follows (Blażejczyk 1994, 2005):

18 $$\mathrm{Irc}={\mathrm{hc}}^{\prime }/\left[{\mathrm{hc}}^{\prime }+\mathrm{hc}+21.55\cdot {10}^{-8}\cdot {\left(\mathrm{Ta}+273\right)}^3\right]$$

19 $$\mathrm{hc}=\left(0.013\cdot \mathrm{ap}-0.04\cdot \mathrm{Ta}\hbox{--} 0.503\right)\cdot {\left(\mathrm{v}+{\mathrm{v}}^{\prime}\right)}^{0.4}$$

20 $${\mathrm{hc}}^{\prime }=\left(0.013\cdot \mathrm{ap}-0.04\cdot \mathrm{Ta}-0.503\right)\cdot 0.53/\left\{{\mathrm{I}}_{\mathrm{cl}}\cdot \left[1-0.27\cdot {\left(\mathrm{v}+{\mathrm{v}}^{\prime}\right)}^{0.4}\right]\right\}$$

*The long-wave* (*L*) *radiation balance* consists of radiation emitted by the surface of the body/clothing (Ls), thermal radiation emitted by the ground surface (Lg), and the reverse radiation of the atmosphere (La):

21 $$\mathrm L=\left(0.5\cdot\mathrm L\mathrm g+0.5\cdot\mathrm L\mathrm a-\mathrm{Ls}\right)\cdot\mathrm{Irc}$$

22 $$\mathrm{Ls}=5.38\cdot10^{-8}\cdot\left(273+\mathrm{Tsk}\right)^4.$$

where Tsk is calculated by the empirical formula below (Blażejczyk 1994, 2005):

23 $$\mathrm{Tsk}=\left(26.4+0.0214\cdot\mathrm M\mathrm{rt}+0.2095\cdot\mathrm{Ta}-0.018\cdot\mathrm{RH}-0.01\cdot\mathrm v\right)+0.6\cdot\left(\mathrm{Icl}-1\right)+0.00128\cdot\mathrm M$$

and:

24 $$\mathrm{Mrt}=\left[\left(\mathrm R/\mathrm{Irc}+\mathrm{Lg}+\mathrm{La}\right)/\left(5.38\cdot10^{-8}\right)\right]^{0.25}-273$$

### Convective heat exchange (C)

Convective heat exchange (C) depends on the difference between the mean skin temperature (Tsk) and the air temperature (Ta), on the speed of air movement, and on its density and heat capacity (represented by hc coefficient):

25 $$\mathrm C=\mathrm{hc}\cdot\left(\mathrm{Ta}-\mathrm{Tsk}\right)\cdot\mathrm{Irc}$$

### Evaporative heat loss (E)

Evaporative heat loss (E) depends on the difference in water vapor pressure on the surface of the skin (vps) and in the ambient air (vp), coefficient of heat transfer by evaporation (he), degree of skin wettedness (w), and dimensionless coefficient of the attenuation of heat flow through the clothing (Ie). According to Fanger (1970), the metabolic heat production (M) which accelerates sweating is also taken into account:

26 $$\mathrm E=\mathrm{he}\cdot\left(\mathrm{vp}-\mathrm{vps}\right)\cdot\mathrm w\cdot\mathrm{Ie}-\left[0.42\cdot\left(\mathrm M-58\right)-5.04\right]$$

27 $$\mathrm{where}\ \mathrm{vps}={\mathrm{e}}^{\left(\mathrm{0,058}\cdot \mathrm{Tsk}+\mathrm{2,003}\right)}$$

28 $$\mathrm{vp}=6.112\cdot {10}^{\left[7.5\cdot \mathrm{Ta}/\left(237.7+\mathrm{Ta}\right)\right]}\cdot 0.01\cdot \mathrm{RH}$$

29 $$\mathrm{w}=1.031/\left(37.5-\mathrm{Tsk}\right)\hbox{--} 0.065$$

30 $$\mathrm{he}=\left[\mathrm{Ta}\cdot \left(0.00006\cdot \mathrm{Ta}-0.00002\cdot \mathrm{ap}+0.011\right)+0.02\cdot \mathrm{ap}-0.773\right)\Big]\cdot 0.53/\left\{\mathrm{Icl}\cdot \left[1-0.27\cdot {\left(\mathrm{v}+{\mathrm{v}}^{\prime}\right)}^{0,4}\right]\right\}$$

31 $$\mathrm{Ie}={\mathrm{hc}}^{\prime }/\left({\mathrm{hc}}^{\prime }+\mathrm{hc}\right)$$

### Heat loss by respiration (Res)

In high mountains, the Res flux is influenced by the lung ventilation volume (Vv), which increases with altitude, both during sleep/relaxation and climbing phases. As altitude increases, the same level of exertion requires increasing minute ventilation (Ve). The method of Cain et al. (1999), which is suggested by Cramer and Jay (2018), was used to estimate the Res flux.

32 $$\begin{aligned}\mathrm{Res}&=\left\{\left[\uprho \cdot \mathrm{cp}\cdot \mathrm{Vv}\cdot \left(\mathrm{Ta}-\mathrm{Tex}\right)+\uprho \cdot \mathrm{Vv}\cdot \left({\mathrm{d}}_{\mathrm{ex}}-{\mathrm{d}}_{\mathrm{a}}\right)\right.\right.\\&\cdot\left.\left. {\mathrm{h}}_{\mathrm{fg}}+\uprho \cdot \mathrm{Vv}\cdot \mathrm{cp}\mathrm{v}\cdot \left(\mathrm{Ta}-\mathrm{Tex}\right)\right]/1000\right\}/\mathrm{Adu}\end{aligned}$$

33 $$\rho=\mathrm{ap}/\left[\left(\mathrm{Ta}+273\right)\cdot287.058\right]$$

34 $${\mathrm{d}}_{\mathrm{a}}=216.7\cdot \mathrm{vp}\cdot {\left(\mathrm{Ta}+273\right)}^{-1}$$

35 $${\mathrm{d}}_{\mathrm{ex}}=216.7\cdot 4.11\cdot {\left(\mathrm{Tex}+273\right)}^{-1}$$

36 $${\mathrm h}_{\mathrm{fg}}=\left[1.005\cdot\mathrm{Ta}+\left(\mathrm{RH}\cdot0.01\right)\cdot\left(2500+\left(1.9\cdot\mathrm{Ta}\right)\right)\right]/1000$$

Vv used in Eq. (32) is calculated as follows:

37 $$\mathrm{Vv}=\mathrm{Ve}/1000\ast 60$$

According to Forte et al. (1997), in the absence of physical effort, the volume of minute ventilation (Ve) increases from about 16 l∙min^−1^ at a pressure of 450 hPa to about 40 l∙min^−1^ at a pressure of 330 hPa. Thus, $\dot{\mathrm{Ve}}$ can be assessed as follows:

38 $$\mathrm{Ve}=108.25-0.2056\cdot\mathrm{ap}.$$

West et al. (1983) have assessed the minute ventilation ($\dot{\mathrm{Ve}}$) during climbing at the altitudes of 6300, 8050, and 8848 m at the VO_2_max level appropriate for a given altitude. Thus, in the current study, Ve is a function of VO_2_ prevailing at different altitudes and of the air pressure itself as follows:

39 $$\mathrm{Ve}=143.932-0.423515\cdot\mathrm{ap}+8.11984\cdot{\mathrm{VO}}_2$$

### Heat exchange by conduction (Cd)

The intensity of heat loss by conduction (Cd) occurring between the human body and ground/snow surface and outer clothing layer is a function of skin-to-ambient temperature difference (ISO 7933):

40 $$\mathrm{Cd}=\mathrm{hdk}\cdot \left(\mathrm{Ta}-\mathrm{Tsk}\right)\cdot {\mathrm{f}}_{\mathrm{cl}}\cdot \mathrm{ca}$$

41 $${\mathrm{f}}_{\mathrm{cl}}=\left(1+1.81\cdot {\mathrm{I}}_{\mathrm{cl}}\right)$$
