# Supplementary material for: Simulations of the human heat balance during Mount Everest summit attempts in spring and winter
Source: Int J Biometeorol. 2023 Dec 19;68(2):351–66. doi: 10.1007/s00484-023-02594-1 (PMC10794380; doi:10.1007/s00484-023-02594-1)
Supplement: Supplementary file 1 — (DOCX 890 kb) [file 484_2023_2594_MOESM1_ESM.docx]

Enlargement of paragraph 4.1.,

Choosing the optimal weather window (continuation of discussion)

# The negative correlation between v and ap observed in May (1979-2019) (Szymczak et al. 2021a) and during 210 non-winter ascents on the summit of Everest (Szymczak et al. 2021b) suggests that with the forecast of lower v climbers might expect higher ap. Higher ap determines higher $\dot{\mathbf{V}}\mathbf{O}_{\mathbf{2}}\mathbf{max}$ and lower minute ventilation at a given altitude, and therefore correspondingly higher speed of ascent, higher M heat gain and lower Res heat losses. Moore and Semple (2011) similarly observed that low ap is typically associated with high v and low Ta. The strong positive correlation between ap and Ta found in many high-altitude studies (Moore and Semple 2011; Matthews et al. 2020a) means that in conditions of higher ap, climbers should expect also higher Ta, and thus a lower C heat losses. In spring the lower risk of hypothermia comes together with lower risk of acute altitude illness (AAI). It may explain why using the wind forecasts alone might increase the chance of reaching the summit and reduce the risk of death during the spring summit ascents.

In winter, the relationship between v, ap and Ta on the summit of Everest changes unfavorably. There is a moderate positive correlation between v and Ta which might mean that during the days of a low wind weather window, Ta would also be lower (Szymczak et al. 2021a). This positive correlation is likely related to the high winter subtropical jet stream (STJ) activity over the Himalayas, as described by Pena-Ortiz et al. (2013). The southward shift of STJ from Himalayas might be associated with lower v on Everest but also lower Ta and ap. Matthews et al. (2020a) observed that extremely low ap periods on Everest’s summit in winter were not associated with strong winter winds. All of above suggest that in lower wind weather window in winter on Everest a climber should expect lower ap and Ta, thus have higher risk of AAI and reduced climbing speed.

Pena-Ortiz C, Gallego D, Ribera P, Ordonez P, Alvarez-Castro M (2013) Observed trends in the global jet stream characteristics during the second half of the 20th century. J. Geophys. Res. Atmos. 118:2702–2713. doi: 10.1002/jgrd.50305

The other references are listed in the main text.

Table SM1. Characteristics of the meteorological stations installed on Everest by the National Geographic expedition

| **Station** | **Latitude**  **(°N)** | **Longitude**  **(°E)** | **Elevation**  **(m a.s.l.)** | **Period of observations**  **start → end** | **Measured variables** |
| --- | --- | --- | --- | --- | --- |
| **Camp 2** | 27.9810 | 86.9023 | 6464 | 8 May 2019 → 31 Dec 2020 | Ta, RH, v, ap, K_glob_, La, Lg |
| **South Col** | 27.9719 | 86.9295 | 7945 | 21 May 2019 → 31 Dec 2020 | Ta, RH, v*, ap, K_glob_, La, Lg |
| **Balcony** | 27.9826 | 86.9292 | 8430 | 22 May 2019 → 20 Jan 2020 | Ta, RH^#^, v^&^, ap |
| Abbreviations: explanations in Table 1of the text body  Remarks: * - data questionable after 6 Jan 2020; # - data available till 20 Dec 2019; & - data available till 24 Oct 2019 | | | | | |

Table SM2. Summit attempt scheme and used clothing assembles and clothing insulation (I_cl_)

| Phase of daily activity | Clothing and sleeping equipment used by mountaineers | I_cl_ (clo) |
| --- | --- | --- |
| Morning and evening relaxation (6-8 a.m. and 4-10 p.m.) | **Spring:** 1+2+3+4 + floor insulation (mat +7)  **Winter:** 1+2+3+5+ floor insulation (mat +7) | 2.5 (spring)  3.0 (winter) |
| Sleeping (10 p.m.- 6 a.m.) | **Spring:** 1+2+3+7+ floor insulation (mat)  **Winter:** 1+2+3+5+7+ floor insulation (mat) | 5.5 (spring)  6.0 (winter) |
| 8 a.m.-4 p.m. – Climbing ascent from Camp 2 to Camp 3 and from Camp 3 to South Col | **Spring:** 1+2+6  **Winter:** 1+2+3+6  Both seasons:  + alpine boots  + balaclava (Polar 100)  + helmet  + rucksack (35 L) | 4.5 (spring)  5.5 (winter) |
| Summit attempt  24-11 - climbing ascent  11 a.m. – reaching summit  11 a.m.-4 p.m. – climbing descent to South Col | **Spring:** 1+2+3+6  **Winter:** 1+2+3+5+6  Both seasons:  + alpine boots  + balaclava (Polar 100)  + helmet  + rucksack (35 L) | 5.5 (spring)  6.0 (winter) |
| 8 a.m.-4 p.m. – climbing descent from South Col to Camp 2 | **Spring:** 1+2+6  **Winter:** 1+2+3+6  Both seasons:  + alpine boots  + balaclava (Polar 100)  + helmet  + rucksack (35 L) | 4.5 (spring)  5.5 (winter) |

Clothing ensembles and sleeping equipment used by mountaineers:

1 – underwear – panties, socks, T-shirt (light, thermo-active material)

[thickness 1 mm] [layers: 1 on arm and calf]

2 – long-sleeve shirt and pants (light thermo-active material)

[thickness 1 mm] [layers: 1 on arm and calf]

3 – long-sleeve shirt and pants (polar fleece 100)

[thickness 2 mm] [layers: 1 on arm and calf]

4 – jacket (polar fleece 200) [thickness 4 mm] [layers: 1 on arm]

5 – jacket (artificial down – for example primaloft)

[thickness 6 mm] [layers: 3 on arm]

6 – down suit [thickness 8 mm] [layers: 3 on arm and calf]

7 – sleeping bag (1 kg of down) [thickness 15 mm] [layers: 3 on arm and calf]

Source: own derivation


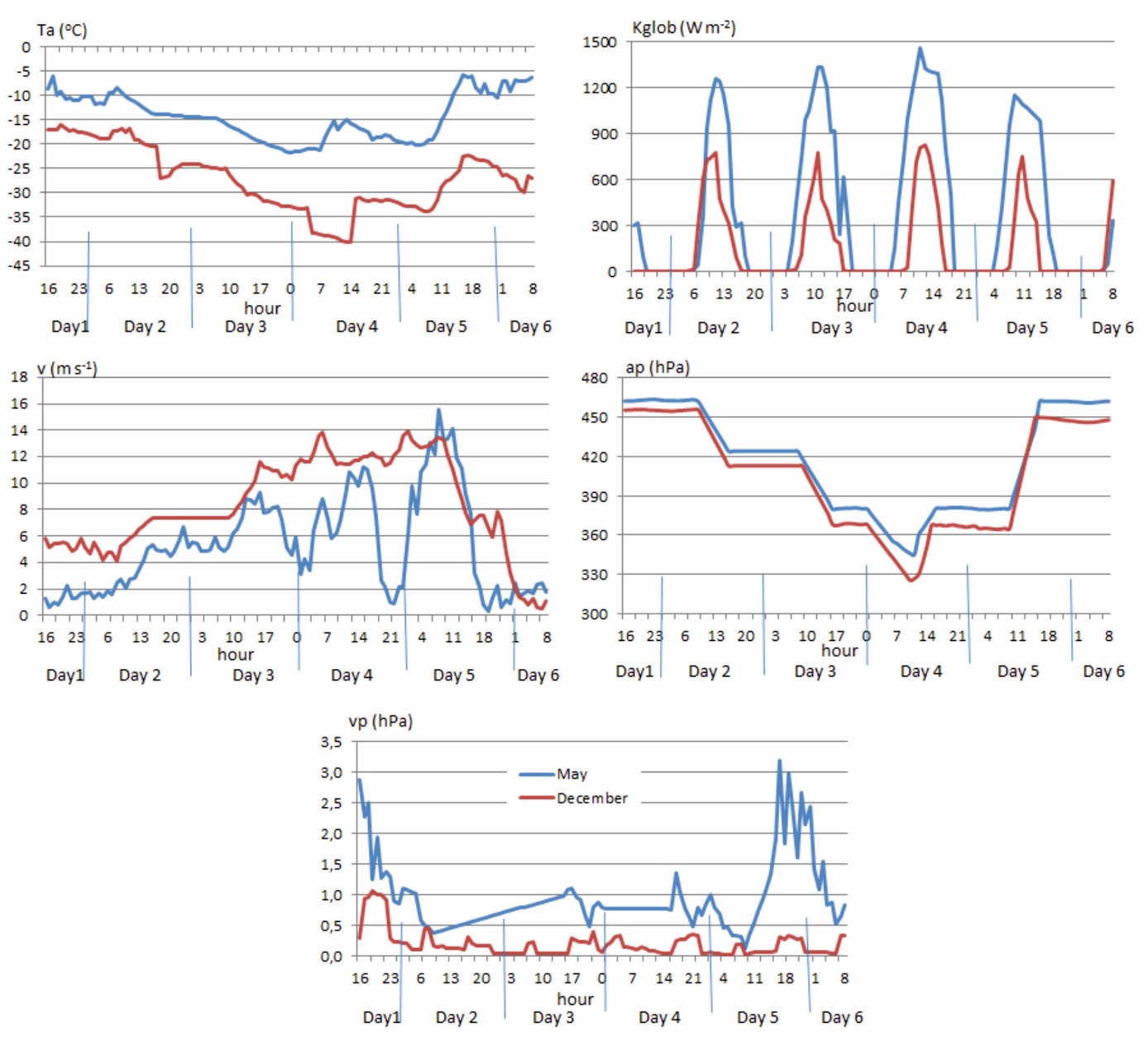


Figure SM1. Values of meteorological elements during days of summit attempts in May and December 2019

Source: Own derivation


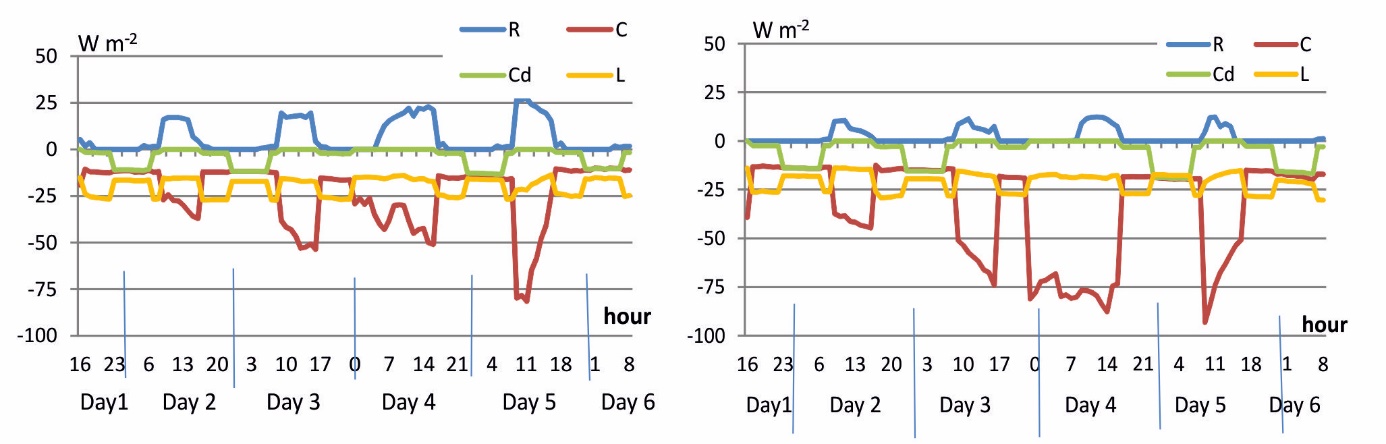


Fig. SM2. Changes in R, Cd, L and C fluxes during summit attempts in May (left panel) and in December (right panel) 2019.

Source: own derivation


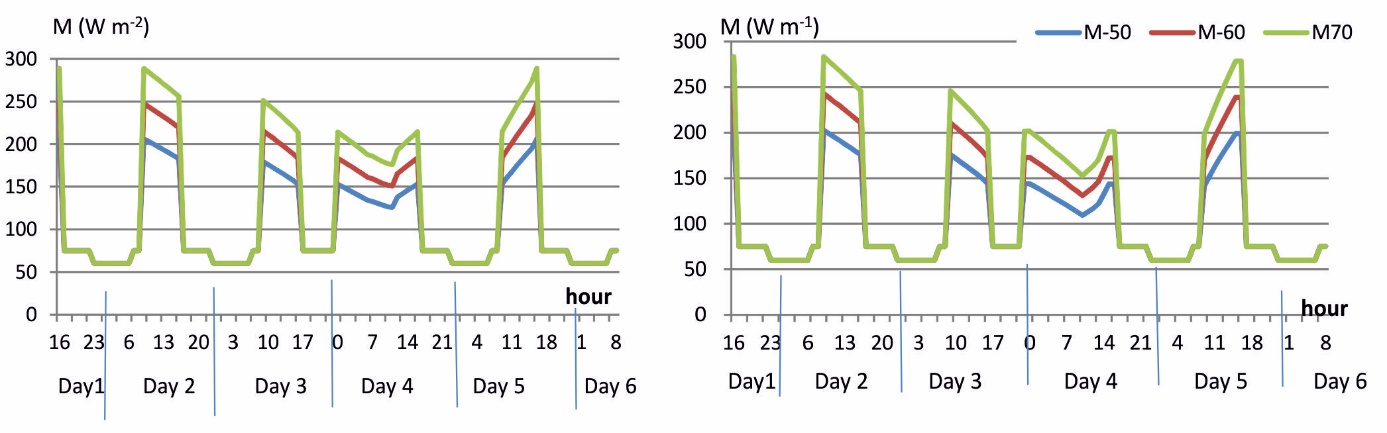


Fig. SM3. Changes in metabolic heat production (M) during summit attempts in May (left panel) and in December (right panel) 2019.

Source: own derivation


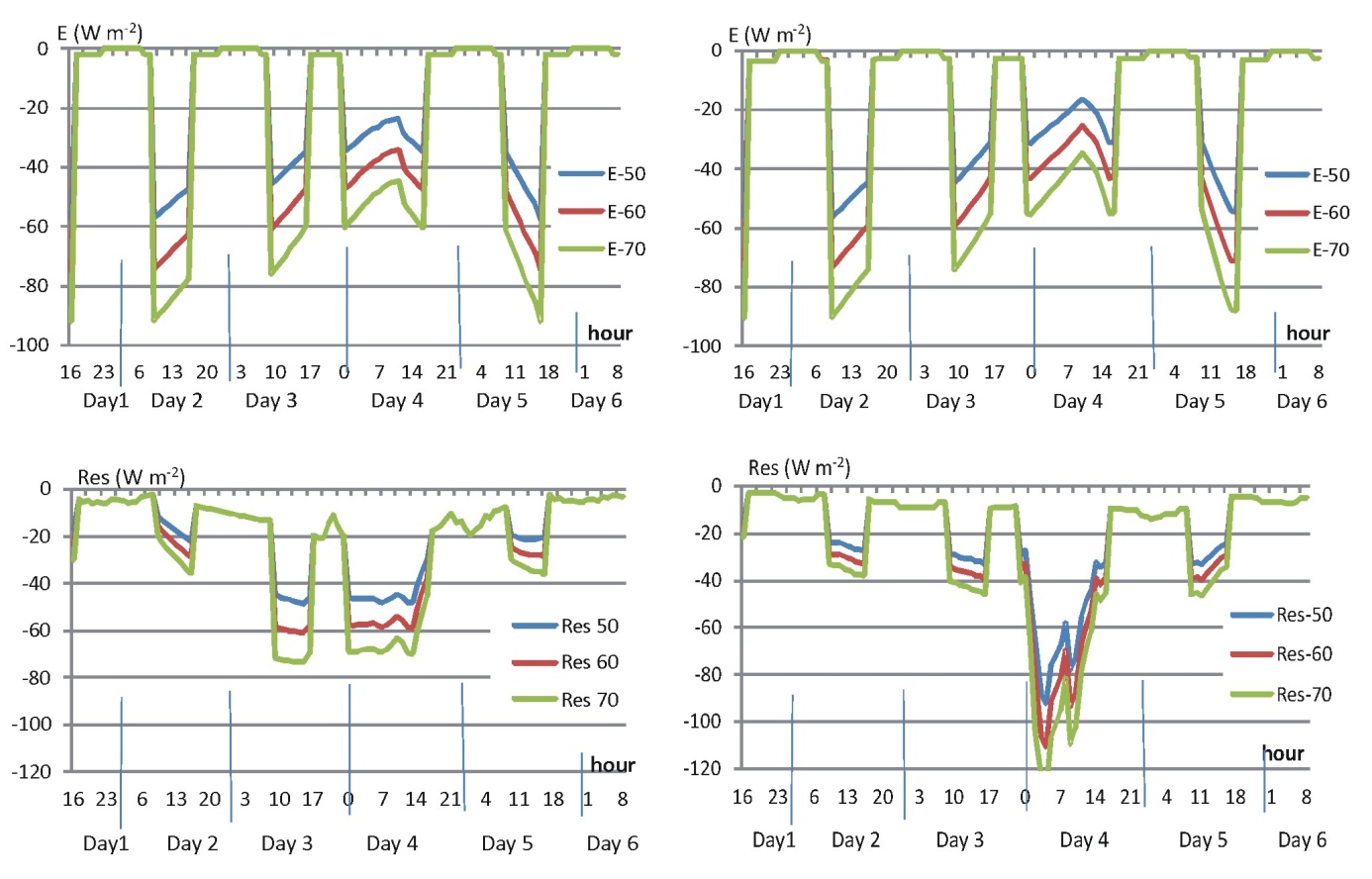


Fig. SM4. Changes in heat loss by evaporation (E) and respiration (Res) during summit attempts in May (left panel) and in December (right panel) 2019.

Source: own derivation
